# The impact of different WHO reference criteria for semen analysis in clinical practice: Who will benefit from the new 2021 thresholds for normal semen parameters?

**DOI:** 10.1111/andr.13213

**Published:** 2022-06-30

**Authors:** Luca Boeri, Giuseppe Fallara, Edoardo Pozzi, Federico Belladelli, Christian Corsini, Massimiliano Raffo, Nicolò Schifano, Paolo Capogrosso, Alessia d'Arma, Francesco Montorsi, Andrea Salonia

**Affiliations:** ^1^ Department of Urology Foundation IRCCS Ca’ Granda, Maggiore Policlinico Hospital Milan Italy; ^2^ Unit of Urology, Division of Experimental Oncology URI IRCCS Ospedale San Raffaele Milan Italy; ^3^ Department of Urology and Andrology Ospedale di Circolo and Macchi Foundation Varese Italy

**Keywords:** classification, male factor infertility, semen parameters, World Health Organization

## Abstract

**Background:**

In 2021, the World Health Organization (WHO) has provided the latest update on processing and evaluating semen analysis.

**Objectives:**

To assess (i) the rate of discordance in semen parameters categorization across three different WHO reference values (namely WHO21, 2010 and 1999) and (ii) the clinical differences among discordant semen analyses from a cohort of primary infertile men.

**Materials and methods:**

Data from 788 infertile men were analyzed. Semen parameters were interpreted based on WHO21, WHO10, and WHO99 reference criteria. Pregnancy outcomes with assisted reproductive techniques (ART) were available for 110 (14%) patients. Descriptive statistics was applied to describe potential differences among the three consecutive WHO references criteria.

**Results:**

Semen parameters categorizations were highly different across the three groups (*p* < 0.001). Of all, 271 (42.2%) patients had normal semen parameters according to WHO10 but were pathologic when considered with WHO21 reference criteria (namely, men with increased semen abnormalities). Infertile men with increased semen abnormalities had lower testicular volume (*p* < 0.001) but higher FSH (*p* < 0.01) and LH (*p* < 0.001) values than those who had no change in terms of semen parameters categorization. Negative ART outcomes were more frequently reported in men with worsening semen parameters compared with those with confirmed semen parameters at WHO21 versus WHO10 (26.8% vs. 49%, *p* = 0.03). Conversely, infertile men with worsening semen parameters at WHO21 versus WHO99 were similar in terms of clinical and hormonal characteristics compared with those with the same rate of semen abnormalities.

**Conclusions:**

One out of three infertile men showed worsened semen categorization according to WHO21 versus WHO10. Infertile men with worsening of semen parameters had worse clinical and hormonal characteristics than those with confirmed numbers of semen abnormalities. Moreover, live birth rates were lower in men with worsening semen abnormalities as for WHO21.

## 1 INTRODUCTION

In Western countries infertility touches about 15% of couples of reproductive age, with a male factor (MFI) present in half of the cases.^1^ In this context, trends in semen quality showed a steep decline of semen concentration among men over the last four decades, parallel with an increased number of in vitro fertilization cycles.^2^, ^3^, ^4^ Moreover, as fertility rates fall and age at the time of the first parenting search rises,^5^ the incidence of MFI and the need for infertility services will likely continue to increase. Therefore, current Guidelines mandate a focused diagnostic work‐up of both partners of infertile couples.^1^, ^6^, ^7^, ^8^ For males, this should include a medical and reproductive history, a physical examination, hormonal investigation and semen analysis, with adherence to World Health Organization (WHO) reference values.^1^, ^7^, ^9^

Although the individual semen parameter provides only a partial indication of actual fertility potential and semen characteristics are not a direct and consequential expression of fertility,^10^, ^11^ routine semen analysis is a key step for MFI investigation, since it provides valuable information about testicular function and it is related to conception chance.^12^ Moreover, poor semen quality, and MFI per se, have been associated with overall man's health and the risk of developing further comorbid disease later in life.^13^, ^14^, ^15^

As for semen analysis, there is a substantial overlap of semen evaluation between fertile and subfertile men,^10^, ^11^ and a substantial variability exists within and among individuals mostly because of cultural, environmental, genetic factors and laboratory techniques.^11^ To this aim, the WHO Laboratory Manual for the Examination and Processing of Human Semen provides standard laboratory methods for semen analysis that are extensively used both in clinical practice and for research purposes. Of clinical importance, the 6th edition of the WHO manual^16^ was published on July 2021 and reports some differences compared with the previous edition (5th edition.) that has been used throughout the last eleven years.^11^

Indeed, in the 5th edition, the distribution of values from approximately 1800 men who have contributed to a natural conception within 12 months of trying was presented and the lower fifth percentile of this distribution has been considered as a true threshold limit for normal versus abnormal semen parameters.^9^ In the 6th edition of the WHO Manual, data from the 5th edition have been further evaluated and complemented with data from around 3500 more men in 12 countries.^17^ Of note, slight differences in reference values (lower 5th percentile) compared with the previous edition was noted.^11^, ^16^

While evidence from both the WHO manual itself and the clinical practice highlights that the lower 5th percentile of data from men in the reference population does not represent a limit between being fertile versus infertile,^11^, ^18^ the segregation of semen parameters as normal versus abnormal (according to the 5th percentile) is still of paramount clinical relevance in the everyday management work‐up. In fact, according to current Guidelines, semen quality severity is considered to guide the indication for diagnostic tests and to suggest potential treatment options for MFI.^1^, ^6^, ^19^

In this context, the clinical impact of currently suggested changes of thresholds for normalcy in terms of pathological semen analysis has never been investigated. Therefore, we aimed to assess (i) the rate of discordance in semen parameters categorization across three different WHO reference values (namely 2021, 2010 and 1999) and (ii) clinical differences among discordant semen analyses from a cohort of non‐Finnish white‐European men consecutively presenting for primary couple's infertility.

## 2 METHODS

We retrospectively reviewed data from a cohort of 1018 non‐Finnish, white‐European men consecutively assessed at a single academic center for primary couple's infertility associated with pure MFI between September 2012 and September 2021. Infertility was defined as not conceiving a pregnancy after at least 12 months of unprotected intercourses regardless of whether or not a pregnancy ultimately occurs.^20^ Patients were only enrolled if they were ≥18 and ≤50 years old and had pure MFI, defined after a comprehensive diagnostic evaluation of all the female partners,^8^ which included a detailed medical, reproductive and family history as well as a general and gynecological physical examination. Furthermore, the ovulatory status, ovarian reserve testing, the structure and patency of the female reproductive tract were requested in all cases.^21^

All participants were assessed with a detailed medical history. We used the Charlson Comorbidity Index (CCI) to score health‐significant comorbidities^22^, ^23^; weight and height were measured and the body mass index (BMI) was calculated for each participant. Testis volume (TV) was assessed using Prader's orchidometer estimation; for the specific purpose of this study we calculated the mean value between the two sides.^24^ Varicocoele was also clinically assessed in every man.^7^ Smoking habit was investigated according to the pack‐year history and then categorized into two groups, as follows: no smokers/former smokers, active smokers.^25^ Duration of infertility and partner's age were collected in every participant.^26^

Venous blood samples were drawn from each patient between 7 AM and 11 AM after an overnight fast. Follicle‐stimulating hormone (FSH), luteinizing hormone (LH), total testosterone (tT), sex hormone‐binding globulin (SHBG), estradiol (E_2_) and prolactin levels were measured for every individual. Chromosomal analysis and genetic testing were performed in every man (karyotype analysis and tests for Y‐chromosome microdeletions and cystic fibrosis mutations).^27^

Participants underwent at least two consecutive semen analyses.^1^, ^7^, ^9^ As for clinical practice, we considered semen volume, sperm concentration, total sperm motility and normal morphology. Semen parameters were interpreted based on 2021, 2010, and 1999 WHO reference criteria (Table 1).

**TABLE 1:** Reference values for semen parameters according to different editions of the WHO Manual for the Examination and Processing of Human Semen

Semen characteristics	WHO 1999	WHO 2010	WHO 2021
Volume (ml)	≥2	1.5	1.4
Sperm concentration (10^6^/ml)	≥20	15	16
Total motility (%)	≥50	40	42
Normal morphology (%)	14	4	4
Normozoospermia 1 semen abnormality 2 semen abnormalities 3 semen abnormalities	31 (3.9%) 217 (27.5%) 293 (37.2%) 247 (31.3%)	138 (17.5%) 269 (34.1%) 235 (29.8%) 146 (18.5%)	126 (16.0%) 257 (32.6%) 238 (30.2%) 167 (21.2%)

*Note*: Prevalence of semen abnormalities according to different reference values in the whole cohort (*n* = 788)

Sperm DNA Fragmentation (SDF), measured by Sperm Chromatin Structure Assay (SCSA), was tested in every participant from January 2015 and it was considered pathological for SDF > 30%.^7^, ^28^ The same laboratory was used for the analysis of all parameters.

We excluded 230 men because they missed one or more of the entry criteria (azoospermia [*n* = 201; 19.7%]; symptoms suggestive for genitourinary infections [*n* = 18; 2.1%]; a history of vasectomy or infertility treatment in the preceding year [*n* = 10; 1.2%]; and partial or incomplete data concerning one or more of the semen parameters considered [*n* = 15; 1.8%]). A convenient sample of 788 infertile men was considered for the statistical analyses.

Pregnancy outcomes with assisted reproductive techniques (ART), defined as live birth rates, were available for 110 (14%) patients.

Data collection followed the principles outlined in the Declaration of Helsinki. All men signed an informed consent agreeing to share their own anonymous information for future studies. The study was approved by the IRCCS San Raffaele Hospital Ethical Committee (Prot. 2014—Pazienti Ambulatoriali).

### 2.1 Statistical methods

**FIGURE 1:**
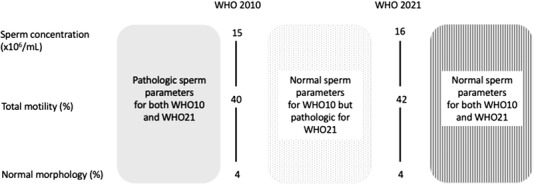
Different categorization of semen parameters according to WHO 2021 versus WHO 2010 reference criteria

Distribution of data was tested graphically and with the Shapiro–Wilk test. Data are presented as medians (interquartile range; IQR) or frequencies (proportions). First, semen parameters were interpreted based on 2021 (WHO21), 2010 (WHO10), and 1999 (WHO99) WHO reference criteria and the rate of discordance in terms of semen abnormalities categorization among groups was investigated. Second, the Kruskal–Wallis test and the Fisher exact were used to investigate demographics, clinical and laboratory characteristics between men who showed increased semen abnormalities when considering WHO21 versus WHO10 and those who had no change in semen parameters categorization. Specifically, the newly proposed thresholds for semen abnormalities according to WHO21 identified a group of men who had normal semen parameters according to WHO10 but were pathologic when considered with WHO21 reference criteria (namely, men with increased semen abnormalities) (Figure 1).

**FIGURE 2:**
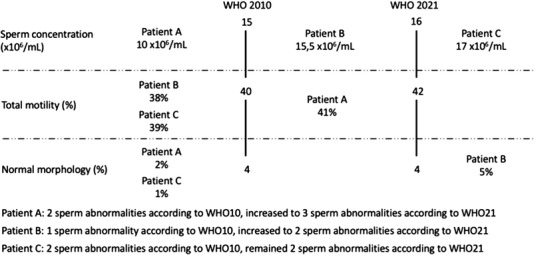
Examples of participants with increased semen abnormalities when considering WHO 2021 reference criteria versus WHO 2010

Similarly, we applied descriptive statistics to compare clinical characteristics and laboratory values between participants who have depicted different types of semen worsening by using WHO21 reference criteria compared with those with no change in semen categorization (Figure 2). Third, we tested the potential difference of clinical characteristics between men who showed increased semen abnormalities when considering WHO99 versus WHO21 and those who had no change in semen parameters categorization. Statistical analyses were performed using SPSS v.26 (IBM Corp., Armonk, NY, USA). All tests were two sided, and statistical significance level was determined at *p* < 0.05.

## 3 RESULTS

Table 1 details the reference values for semen parameters according to different editions of the WHO Manual for the Examination and Processing of Human Semen. Moreover, the prevalence of semen abnormalities according to different reference values in the whole cohort was also reported.

**FIGURE 3:**
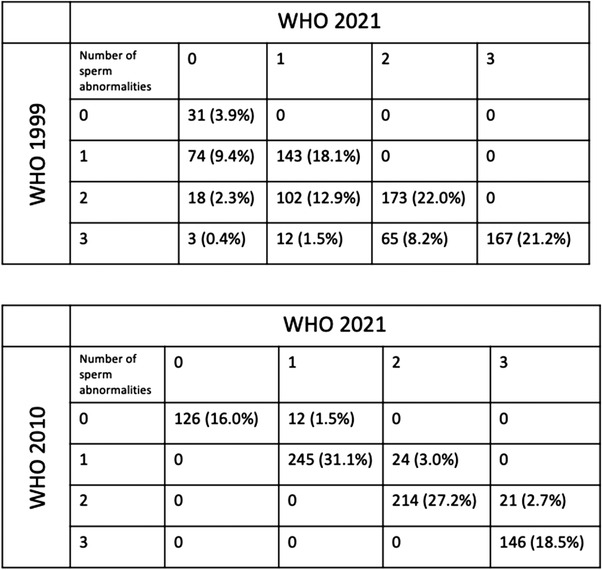
Rates of semen alterations according to different reference criteria as for WHO 2021, WHO 2010, and WHO 1999

Semen parameters categorizations were highly different across the three groups (*p* < 0.001) (Figure 3). Overall, normozoospermia was found in 31 (3.9%), 138 (17.5%), and 126 (16.0%) patients according to WHO99, WHO10, and WHO21, respectively (*p* < 0.001). Similarly, oligoasthenoteratozoospermia was reported in 247 (31.3%), 146 (18.5%), and 167 (21.2%) men according to WHO99, WHO10, and WHO21, respectively (*p* < 0.001).

Of 788, 271 (34.4%) men had increased semen abnormalities when considering WHO21 versus WHO10.

Tables 2 and 3 report descriptive statistics of participants who had increased semen abnormalities by WHO21 compared with those whose semen parameters did not change. Infertile men with increased semen abnormalities had lower TV (*p* < 0.001), higher FSH and LH values (all *p* ≤ 0.01) than those who did not depict change in semen parameters categorization. A higher rate of genetic alterations (any type) was found in men with worsening semen categorization (*p* = 0.02) (Table 3). Of note, SDF was higher (*p* < 0.001) in men with worsening of their categorization according to WHO21 versus WHO10. Likewise, a greater prevalence of men had SDF > 30% after worsening of semen parameters categorization at WHO21 compared with WHO10 (*p* < 0.001). Of note, we found that 12.3% versus 11.9% (*p* = 0.6) men with unexplained infertility had abnormal SDF by WHO10 and WHO21, respectively.

**TABLE 2:** Descriptive statistics of participants as segregated according to worsening semen categorization by using WHO21 versus WHO10 reference criteria (no. = 642*)

	Overall	No change in semen abnormalities	Increased number of semen abnormalities	*p* Value^§^
No. of patients (no. [%])	642 (100)	371 (57.8)	271 (42.2)	
Age (years)				0.2
Median (IQR)	37.0 (33–41)	36.0 (33–40)	37.0 (33–41)	
Range	20–50	20–50	20–50	
BMI (kg/m^2^)				0.5
Median (IQR)	24.8 (23.1–26.7)	24.7 (23.1–26.5)	24.9 (23.1–26.7)	
Range	19.9–41.2	20.0–37.6	19.9–41.2	
CCI (value)				0.9
Median (IQR)	0.0 (0.0)	0.0 (0.0)	0.0 (0.0)	
Mean (SD)	0.08 (0.4)	0.09 (0.4)	0.07 (0.5)	
Range	0–3	0–3	0–3	
CCI ≥1 (no. [%])	14 (2.1)	7 (1.8)	7 (2.5)	0.3
Partner's age (years)				0.4
Median (IQR)	34.0 (32–38)	34.0 (32–38)	34.0 (31–38)	
Range	20.0–48.0	20.0–48.0	20.0–47.0	
Duration of infertility (months)				0.5
Median (IQR)	18.0 (12–30)	18.0 (12–26)	19.0 (12–30)	
Range	12.0–60.0	12.0–60.0	12.0–58.0	
Testis volume (Prader estimation)				<0.001
Median (IQR)	18.0 (14–20)	20.0 (15–25)	15.0 (12–20)	
Range	6–25	6–25	6–25	
Clinical varicocoele (no. [%])	364 (56.6)	207 (55.8)	157 (57.9)	0.6
History of cryptorchidism (no. [%])	55 (8.5)	28 (7.6)	27 (10.3)	0.2
Genetic alterations (any type) (no. [%])	47 (7.3)	20 (5.4)	27 (10.0)	0.02
Current smoking status (no. [%])	211 (32.8)	121 (32.7)	90 (33.4)	0.5

* After excluding 146 participants that showed 3 semen abnormalities according to both WHO10 and WHO21.

^§^ *p* Value according to the Mann–Whitney test for continuous data and the Fisher exact test for categorical variables, as indicated.

Abbreviations: BMI = body mass index; CCI = Charlson Comorbidity Index.

**TABLE 3:** Descriptive statistics of participants as segregated according to worsening semen categorization by using WHO21 versus WHO10 reference criteria (no. = 642*)

	Overall	No change in semen abnormalities	Increased number of semen abnormalities	*p* Value^§^
FSH (mUI/ml)				<0.001
Median (IQR)	4.3 (2.9–7.1)	3.7 (2.7–6.0)	5.0 (3.4–9.1)	
Range	0.6–32.7	0.6–21.8	0.7–32.7	
LH (mUI/ml)				<0.001
Median (IQR)	3.8 (2.7–5.1)	3.6 (2.4–4.7)	4.2 (3.0–5.4)	
Range	0.3–16.0	0.3–13.3	0.9–16.0	
Total testosterone (ng/ml)				0.1
Median (IQR)	4.7 (3.6–5.8)	4.8 (3.6–5.7)	4.7 (3.5–5.9)	
Range	0.9–20.6	0.9–20.6	1.2–15.5	
SHBG (nmol/L)				0.3
Median (IQR)	36.0 (27–45)	35.0 (27.0–44.1)	36.0 (27.6–46)	
Range	7.5–154.0	11.0–154.0	7.5–135.0	
E_2_ (pg/ml)				0.6
Mean (SD)	25.0 (27–45)	25.0 (24–34)	24.9 (21–38)	
Range	1.0–89.6	1.0–89.6	2.2–78.2	
Inhibin B (pg/ml)				0.02
Median (IQR)	144.6 (104.1–205.8)	151.6 (115.8–211.7)	132.7 (78.6–195.7)	
Range	5.2–671.3	14.0–538.0	5.2–671.3	
Prolactin (ng/ml)				0.1
Median (IQR)	8.5 (6.5–11.9)	8.2 (6.2–10.7)	9.1 (6.8–12.4)	
Range	1.9–67.7	1.9–45.7	2.1–67.7	
Semen volume (ml)				<0.001
Median (IQR)	3.0 (2–4)	3.0 (2–4)	3.0 (2–4)	
Range	0.9–10.0	0.9–10.0	0.5–9.0	
Sperm concentration (×10^6^/ml)				<0.001
Median (IQR)	24.0 (8.5–50.0)	38.3 (20.0–65.1)	9.0 (3.5–22.0)	
Range	0.1–455.3	0.5–455.3	0.1–114.0	
Total motility (%)				<0.001
Median (IQR)	50.0 (37–62)	55.0 (46–67)	40.0 (30–50)	
Range	0.0–122.0	0.0–122.0	0.0–100.0	
Normal morphology (%)				<0.001
Median (IQR)	3.0 (1–10)	5.0 (2–14)	2.0 (1–5)	
Range	0.0–100.0	0.0–94.0	0.0–100.0	
SDF (%)	*n* = 270	*n* = 140	*n* = 130	<0.001
Median (IQR)	25.7 (16.2–42.2)	23.0 (13.6–35.0)	30.8 (19.3–49.7)	
Range	0.4–96.4	0.4–90.4	1.5–96.4	
SDF > 30% (no. [%])	119 (44.1)	44 (31.4)	75 (56.9)	<0.001
Assisted‐pregnancy rate (no. [%])	36 (39.1)	25 (49.0)	11 (26.8)	0.03
*n* = 92				

Abbreviations: FSH = follicle stimulating hormone; LH = luteinizing hormone; SHBG = sex hormone binding globulin; E_2_ = estradiol; SDF = sperm DNA fragmentation index.

* After excluding 146 participants that showed 3 semen abnormalities according to both WHO10 and WHO21.

^§^ *p* Value according to the Mann–Whitney test for continuous data and the Fisher Exact Test for categorical variables, as indicated.

Negative ART outcomes were more frequently reported in men with worsening semen parameters than those with confirmed semen parameters categorization at WHO21 versus WHO10 (*p* = 0.03) (Table 3).

Supplementary Tables 1 and 2 depict clinical characteristics of participants according to different type of semen parameters categorization. In this context, patients with 2 semen abnormalities at WHO10 that increased to 3 semen abnormalities at WHO21 showed lower TV (*p* < 0.01), but higher FSH values (*p* = 0.03) and SDF (*p* = 0.03) than those with 2 semen abnormalities both at WHO21 and WHO10. Of note, clinical characteristics of men who increased from 2 to 3 semen abnormalities were similar to those of men with confirmed 3 abnormalities at both editions of the manual.

No differences were found among men who had increased from 1 to 2 semen abnormalities at WHO21 versus WHO10 compared with those with confirmed either 1 or 2 semen abnormalities at both editions. Similarly, infertile men with worsening semen parameters categorization by using WHO99 versus WHO21 depicted similar clinical, hormonal and SDF characteristics compared with those with the same rate of semen abnormalities at both editions.

## 4 DISCUSSION

Semen analysis is the backbone of the diagnostic work‐up of each infertile male.^1^, ^7^ Thereof, decision making for the clinical management of MFI is often based on semen quality and the severity of semen abnormalities.^1^ Recently, the 2021 edition of the WHO manual for the examination of semen samples has proposed new reference values for semen abnormalities that differed from those of the latest 2010 edition.^11^ To the best of our knowledge an explorative investigation of the clinical impact of this change in terms of reference values of semen parameters is currently lacking.

Here, we found that one out of three infertile men showed increased severity of sperm categorization according to WHO21 versus WHO10, thus meaning that semen parameters were above the reference limit for normality according to WHO10 while they have been categorized as pathologic according to the new WHO21 reference limits. Infertile men with worsening sperm categorization had worse clinical, hormonal, and reproductive parameters as compared with those without changes in semen abnormalities rates. Conversely, this difference was not found when considering WHO99 versus WHO21 reference criteria. Taking together, these findings would suggest that the WHO21 criteria better identify a subgroup of patients with impaired reproductive health despite being considered with normal semen parameters according to WHO10 reference criteria.

It is well known that there is a substantial intersection of semen quality between fertile and subfertile men, and there are no distinct confines between fertile versus infertile men only relying on semen parameters at semen analysis.^10^, ^11^ Nonetheless, semen quality is used in clinical practice to tailor the management work‐up in men presenting for couple's infertility.^7^ For instance, the American Urological Association/American Society for Reproductive Medicine (AUA/ASRM) Guidelines for the diagnosis and treatment of MFI suggest obtaining hormonal evaluation in men with impaired libido, erectile dysfunction, oligozoospermia, or azoospermia.^6^, ^29^ Likewise, genetic testing is advise by the AUA/ASRM and EAU Guidelines in azoospermic men or in infertile men with severe oligozoospermia.^1^, ^6^ In terms of MFI treatment, the use of gonadotropins is considered in men with idiopathic oligozoospermia and FSH values within the normal range to improve spermatogenic outcome.^1^, ^7^ Lastly, the treatment of clinical varicocoele has been suggested in men with abnormal semen parameters and otherwise unexplained infertility,^1^, ^7^ thus highlighting the importance of reference values for considering normal versus impaired semen quality in the everyday clinical practice.

Of clinical importance, the severity of semen abnormalities has been associated with health outcomes in infertile men. Indeed, previous studies have shown that semen parameters were inversely associated with the overall burden of comorbid conditions in infertile men^23^, ^30^, ^31^; similarly, a recent study with 1957 infertile men showed that CCI progressively increased along with the number of semen alterations.^10^ In this context MFI can be considered as a proxy of overall men's health.^32^, ^33^

From a clinical standpoint, semen quality has been associated with the clinical and hormonal profile of infertile men. Boeri et al., for instance, showed that clinical characteristics and hormonal parameters of infertile men got worse as the number of semen alterations increased.^10^ Moreover, TV has been considered a good clinical marker of reproductive function and previous studies have reported the association between a reduced TV and poor semen parameters.^10^ In terms of reproductive outcomes, SDF testing has progressively emerged as an important tool for the clinical management of MFI.^34^, ^35^ Several conditions have been associated with increasing SDF in infertile men, thus including recreational habits, environmental toxins, varicocoele, and genital infections^35^, ^36^; however, the correlation between SDF with semen quality by routine semen analysis is still a matter of debate. In fact, some studies have reported a negative correlation between SDF and semen parameters,^37^, ^38^ but this was not the case for other reports.^39^ Our results are concordant with these findings; indeed, we showed that infertile men with worsening semen parameters categorization had lower TV and higher FSH values than those with confirmed semen abnormalities as for WHO21 and WHO10, respectively. Moreover, SDF and live birth rates were lower in men with worsening semen categorization. In particular, we found that infertile men with 2 semen abnormalities at WHO10 who conversely increased to 3 semen abnormalities at WHO21, actually have clinical and hormonal characteristics similar to men with 3 semen abnormalities for both WHO editions and even worse parameters than those with only confirmed 2 semen abnormalities. These results suggest that the new reference values for semen quality proposed by WHO21 is able to even better identify, and actually categorize as having “pathologic” semen quality, a group of men with impaired clinical characteristics and reproductive parameters despite being considered with normal semen parameters according to WHO10 reference criteria. In light of the known association between semen quality and clinical features and health outcomes, the new classification is therefore more rigorous in recognizing infertile men with impaired semen and health parameters.

Our study is novel since we conducted the first real‐life contemporary investigation of the rate of men with normal and abnormal semen parameters according to different reference values proposed by three WHO editions over a 20 years period in a homogenous, same‐ethnicity, age‐comparable cohort of infertile men. Second, we detailed the importance of the association between semen impairment and patient's characteristics, thus highlighting the value of different reference values for semen analysis.

Likewise, our study is not devoid of limitations. First, this was a single center‐based study, raising the possibility of selection biases; thereof, larger studies are needed to externally validate our findings. Second, despite our evaluation includes a comprehensive assessment of the whole cohort in terms of clinical, semen and hormonal evaluation, we lacked an oxidative stress investigation in the entire cohort of participants, which is known for its negative effect toward semen quality.^40^

## 5 CONCLUSIONS

The recent introduction of new reference criteria for semen parameters by WHO21 resulted in a different categorization of severity of semen abnormalities as compared with both previous editions. Indeed, one out of three infertile men showed worsened semen categorization according to WHO21 versus WHO10. Infertile men with worsening semen parameters had worse clinical, hormonal and SDF characteristics than those with confirmed numbers of semen abnormalities. Moreover, pregnancy outcomes with assisted reproductive techniques were lower in men with worsening semen abnormalities as for WHO21. Overall, WHO21 criteria better identify a subgroup of patients with impaired reproductive health despite being considered with normal semen parameters according to WHO10. According to WHO21 reference criteria, semen quality ought to be considered as a continuum and the artificial binary division between “normal” and “abnormal” should be used cautiously and only considered as a rough guidance in clinical practice. Further studies are needed to externally confirm these observations.

## CONFLICT OF INTERESTS

The authors have declared that no conflict of interest exists

## AUTHOR CONTRIBUTIONS

LB and AS designed and led the study and wrote the report. PC, GF, EP, FB, CC, MR, NS, AD, and FM took care of patients and acquired data. LB and AS analyzed data and drafted the report.

## Supporting information

Supporting Information
