# Supplementary material for: The impact of different WHO reference criteria for semen analysis in clinical practice: Who will benefit from the new 2021 thresholds for normal semen parameters?
Source: Andrology. 2022 Jun 30;10(6):1134–42. doi: 10.1111/andr.13213 (PMC9541878; doi:10.1111/andr.13213)
Supplement: Supplementary file 1 — Supporting Information [file ANDR-10-1134-s001.doc]

**Supplementary table 1: Descriptive statistics of participants as segregated according to worsening semen categorization by using WHO21 vs. WHO10**

**reference criteria**

**2 semen abnormalities 2 semen abnormalities 3 semen abnormalities**

**Increased to 3 remained 2 remained 3 p value^*^**

No. of patients [No. (%)] 21 (2.6) 214 (27.1) 146 (18.5)

Age (years) 0.7

Median (IQR) 36.0 (33-40) 36.0 (33-40) 37.0 (34-40)

Range 20 - 50 20 – 48 20 - 50

BMI (kg/m^2^) 0.8

Median (IQR) 24.7 (23.4-26.5) 25.1 (23.0-26.8) 24.9 (22.9-26.8)

Range 21.0 – 34.0 20.0 – 41.1 19.9 – 35.6

CCI (value) 0.6

Median (IQR) 0.0 (0.0) 0.0 (0.0) 0.0 (0.0)

Mean (SD) 0.14 (0.2) 0.07 (0.2) 0.12 (0.5)

Range 0 – 2 0 – 2 0 – 3

Partner’s age (years) 0.6

Median (IQR) 33.0 (32-36) 34.0 (31-38) 35.0 (32-37)

Range 26.0 – 44.0 21.0 – 47.0 23.0 – 44.0

Duration of infertility (months) 0.3

Median (IQR) 24.0 (12-24) 19.0 (12-36) 18.0 (12-24)

Range 12.0 – 48.0 12.0 – 59.0 12.0 – 60.0

Testis volume (Prader estimation) <0.01

Median (IQR) 12.0 (11-18) ^§^ 15.0 (12-20) 12.0 (11-17) ^§^

Range 5 – 25 6 – 25 6 - 25

Clinical Varicocele [No. (%)] 12 (57.1) 125 (58.4) 94 (64.4) 0.4

History of cryptorchidism [No. (%)] 1 (4.7) 16 (7.4) 17 (11.6) 0.1

Genetic alterations (any type) [No. (%)] 2 (9.5) 23 (10.7) 16 (11.0) 0.9

Current smoking status [No. (%)] 6 (28.6) 55 (25.7) 50 (34.2) 0.2

Keys: BMI = body mass index; CCI = Charlson Comorbidity Index

*P value according to the Kruskal-Wallis test for continuous data and the Fisher Exact Test for categorical variables, as indicated

§ p<0.01 vs. group: 2 semen abnormalities remained 2.

**Supplementary table 2: Descriptive statistics of participants as segregated according to worsening semen categorization by using WHO21 vs. WHO10**

**reference criteria**

**2 semen abnormalities 2 semen abnormalities 3 semen abnormalities**

**Increased to 3 remained 2 remained 3 p value***

FSH (mUI/mL) 0.03

Median (IQR) 6.8 (3.6-9.8) ^§^ 5.0 (3.4-9.1) 6.9 (4.3-10.6) ^§^

Range 1.6 – 16.1 0.5 – 32.7 1.4 – 37.0

LH (mUI/mL) 0.2

Median (IQR) 468 (3.1-5.8) 4.2 (3.1-5.4) 4.7 (3.5-6.0)

Range 2.3 – 9.8 0.9 – 16.0 1.3 – 24.0

Total Testosterone (ng/mL) 0.8

Median (IQR) 4.9 (3.2-6.1) 5.1 (3.5-6.4) 4.6 (3.5-5.8)

Range 2.0 – 15.0 1.2 – 20.6 2.1 – 18.2

SHBG (nmol/L) 0.6

Median (IQR) 39.0 (31-47) 36.0 (27.0-46.3) 35.0 (26.7-43.2)

Range 18.0 – 69.0 14.5 – 135.0 14.3 – 81.0

E_2_ (pg/mL) 0.4

Mean (SD) 24.0 (19-30) 24.0 (22-34) 24.0 (22-32)

Range 2.2 – 40.1 5.0 – 115.0 5.0 – 87.2

Inhibin B (pg/mL) 0.3

Median (IQR) 119.1 (73.2-199.2) 127.9 (73.9-194.7) 117.7 (61.7-167.7)

Range 9.8 – 241.5 5.2 – 538.0 6.0 – 372.7

Prolactin (ng/mL) 0.7

Median (IQR) 7.8 (6.2-13.5) 9.0 (7.0-12.5) 9.1 (7.0-13.0)

Range 4.5 – 26.9 2.0 – 43.7 1.9 – 44.9

Semen volume (mL) 0.6

Median (IQR) 2.5 (2-5) 3.0 (2-4) 3.0 (2-4)

Range 0.5 – 7.5 0.9 – 9.4 0.5 – 7.5

Sperm concentration (x10^6^/mL) <0.001

Median (IQR) 6.0 (1.8-12.7) 8.9 (3.4-22.5) 3.0 (1.0-6.1) ^§^

Range 0.2 – 15.0 0.1 – 114.1 0.1 – 14.2

Total motility (%) <0.001

Median (IQR) 36.0 (28-46) 40.0 (36-51) 22.0 (10-30) ^§^

Range 9.0 – 42.0 0.0 – 100.0 0.0 – 39.0

Normal morphology (%) <0.001

Median (IQR) 1.0 (1-2) 2.0 (1-4) 1.0 (1-2) ^§^

Range 0.0 – 3.0 0.0 – 82.0 0.0 – 3.0

Sperm DNA fragmentation index (%) n=21 n=102 n=80 0.03

Median (IQR) 37.9 (21.3-51.6) ^§^ 31.5 (20.4-50.3) 41.2 (27.8-60.9) ^§^

Range 5.4 – 89.3 1.5 – 96.4 13.1 – 97.8

Sperm DNA fragmentation index >30% [No. (%)] 13 (61.9) 54 (52.9) 54 (67.5) <0.001

n=21 n=56 n=27

Assisted-pregnancy rate [No. (%)] 7 (33.3) 24 (42.8) 9 (33.3) 0.01

Keys: FSH = Follicle Stimulating Hormone; LH = Luteinizing Hormone; SHBG = Sex Hormone Binding Globulin; E_2_ = Estradiol;

* P value according to the Kruskal-Wallis test for continuous data and the Fisher Exact Test for categorical variables, as indicated

§ p<0.01 vs. group 2 semen abnormalities remained 2.
